# Klebsiella pneumoniae primary liver abscess associated with ruxolitinib

**DOI:** 10.1007/s00277-016-2718-7

**Published:** 2016-06-04

**Authors:** Yoshiharu Kusano, Yasuhito Terui, Kyoko Ueda, Kiyohiko Hatake

**Affiliations:** Department of Hematology and Oncology, Cancer Institute Hospital of Japanese Foundation for Cancer Research, 3-8-31, Ariake, Koto-ward, Tokyo, Japan 135-8550

**Keywords:** Primary myelofibrosis, Ruxolitinib, Pyogenic liver abscess

Dear Editor,

Pyogenic liver abscess is an uncommon intra-abdominal infection associated with high mortality. The liver is in contact with massive quantities of blood from systemic and portal circulation. Therefore, any breakdown of its immune system can directly cause lethal infections. Patients with Klebsiella pneumoniae primary liver abscess (KPPLA) have higher incidences of diabetes or glucose intolerance compared to those with other pyogenic liver abscess, but it is unknown how diabetes influences the liver’s immunity including Kupffer’s cells. Here, we present a case with primary myelofibrosis (PMF) suffering from KPPLA during the course of ruxolitinib, a JAK1 and JAK2 inhibitor.

A 78-year-old man had a diagnosis of JAK2V617F+ PMF. Treatment with ruxolitinib 20 mg twice daily was initiated in May 2015. At the 2-month follow-up, he was suffering from herpes zoster and thrombocytopenia, so the dosage of ruxolitinib was reduced to 10 mg twice daily. Since dosage was reduced, absolute hemoglobin and thrombocyte counts had been stable. In January 2016, he presented with fatigue, fever with chills, and persistent right upper quadrant pain. Blood pressure was 91/50 mmHg, pulse was 120 beats per minute, respiratory rate was 26 breaths per minute, and oxygen saturation was 98 % while he was breathing a room air. A laboratory blood test showed elevated biliary and liver enzymes, prothrombin time (PT), activated partial thromboplastin time, and fibrinogen degradation products (FDP), whereas hemoglobin and platelets were decreased due to disseminated intravascular coagulopathy (DIC), which was score 4 based on DIC diagnostic criteria (systemic inflammatory response syndrome, platelet count of 8.5 × 10^4^/μL, FDP > 10 μg/ml, and PT-international normalized ratio > 1.2). The absolute neutrophil count and immunoglobulin levels were normal. Abdominal contrast-enhanced computer tomography scans showed an abscess with a 5-cm radius (Fig. 1). Percutaneous catheter drainage of abscess was performed, and piperacillin-tazobactam 4.5 mg thrice daily was initiated. An alleviation of fever was observed from the next day, and the volume of the abscess dwindled considerably in 14 days. Klebsiella pneumoniae was detected from the pus. Magnetic resonance imaging did not reveal any predisposing intra-abdominal factors for abscess formation. The catheter was removed and the patient was discharged from hospital continuing on cefcapene 100 mg trice daily for another 3 weeks.

**Fig. 1:**
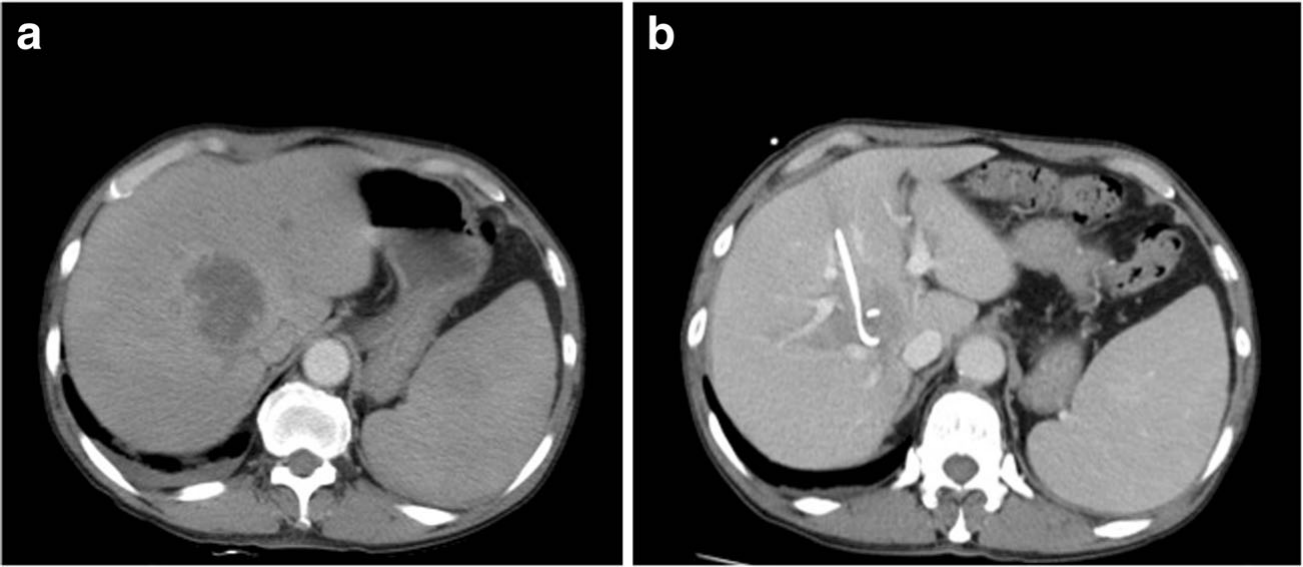
**a** Liver abscess with a 5-cm radius before drainage. **b** Diminished abscess after 14-day catheter drainage

Ruxolitinib interferes with a variety of immune cells and their function [1–3]. In fact, the number and activity of immune cells were diminishing in our case: B cells, 28/μl; CD4 + T cells, 50/μl; CD8+ T cells, 53/μl; and NK-cell activity, 4 % (normal 18–40). With severe impairment of the number and activity of NK cells, JAK mutations and STAT deficiency can cause not only viral infection but also severe bacterial infections [4]. The defect in cell-mediated immunity combined with or without impaired function of B cells might be induced by ruxolitinib, which is thought to be associated with KPPLA.

ECOG, Eastern Cooperative Oncology Group; FDP, fibrinogen degradation products; JAK, Janus kinase; KPPLA, Klebsiella pneumoniae primary liver abscess; PMF, primary myelofibrosis; PT, prothrombin time; STAT, signal transducer and activator of transcription; DIC, disseminated intravascular coagulopathy

## References

[1] Schönberg K, Rudolph J, Vonnahme M, Parampalli Yajnanarayana S, Cornez I, Hejazi M (2015). JAK inhibition impairs NK cell function in myeloproliferative neoplasms. Cancer Res.

[2] Heine A, Held SA, Daecke SN, Wallner S, Yajananayana SP, Kurts C (2013). The JAK-inhibitor ruxolitinib impairs dendritic cell function in vitro and in vivo. Blood.

[3] Parampalli Yajnanarayana S, Steobig T, Comez I, Alchalby H, Scheonberg K, Rudolph J (2015). JAK1/2 inhibition impairs T cell function in vitro and in patients with myeloproliferative neoplasms. Br J Hematol.

[4] O’Shea JJ, Holland SM, Staudt LM (2013). JAKs and STATs in immunity, immunodeficiency, and cancer. N Engl J Med.

